# Prognostication for Body Contouring Surgery After Bariatric Surgery

**Published:** 2012-09-12

**Authors:** Devinder Singh, Antonio J. V. Forte, Hamid R. Zahiri, Lindsay E. Janes, Jennifer Sabino, Jamil A. Matthews, Robert L. Bell, J. Grant Thomson

**Affiliations:** ^a^Division of Plastic Surgery, Department of Surgery, University of Maryland Medical Center, Baltimore, Md; ^b^Yale University School of Medicine, Section of Plastic Surgery, New Haven, Conn; ^c^Yale University School of Medicine, Division of Bariatric Surgery, New Haven, Conn

## Abstract

**Objective:** Body contouring surgery has become a steadily increasing part of weight loss treatment in the population of patients electing to undergo bariatric surgery. This study aims to elicit factors that can be used to prognosticate which bariatric surgery patients will choose to undergo body contouring procedures. **Methods:** A database of 381 patients who underwent gastric bypass surgery between August 2002 and December 2005 was retrospectively reviewed. All patients with subsequent body contouring surgery (group I) were identified and compared with those without it (group II). Variables studied were age, gender, preoperative excess body weight, percent excess weight loss at 6 and 12 months, preoperative body mass index, and change in body mass index at 6 and 12 months. **Results:** We identified 24 patients for group I and 168 patients for group II. Group I was significantly younger with a mean age of 36 ± 9 years than group II with a mean age of 41 ± 10 years (*P* = .023). Change in body mass index was significantly greater in group I with changes of 16.1 ± 4 and 13.82 ± 3 (*P* = .001) at 6 months and changes of 21.4 ± 6.6 and 17.39 ± 4.6 (*P* < .0001) at 12 months in group I and group II, respectively. Lastly, the percent excess weight loss at 12 months was significantly greater in group I with a mean percent excess weight loss of 70.1 ± 13.3 than in group II with a mean percent excess weight loss of 62 ± 16.6 (*P* = .0052). **Conclusions:** Age, change in body mass index at 6 and 12 months, and percent excess weight loss at 12 month follow-up were useful predictive factors to determine which bariatric surgery patients ultimately underwent body contouring procedures.

With 74 million obese US adults, about one fifth of whom are morbidly obese, an increasing number of patients are turning to bariatric surgeons located in centers of excellence.^1^^-^3 These centers house multiple medical specialties allowing for comprehensive care throughout the course of a patient's weight loss. A major impetus for interdisciplinary care is the rapid rate of secondary weight loss that often leads to distressing deformities including excess skin and malpositioned adipose tissue. In addition to a compromised body image, this redundant skin can cause functional impairment by harboring intertriginous rashes or by interfering with ambulation, maintaining adequate hygiene, and activities of daily living.^4^^,^^5^ Dissatisfaction with these postbariatric surgical deformities spurs patients to seek out body contouring procedures. As such, plastic surgeons play a vital role within these centers of excellence for bariatric patients.

However, not every patient who undergoes bariatric surgery will have troublesome skin folds or require plastic surgery. The excessive skin folds can appear anywhere on the body and surgeons lack precision when predicting which deformities may present.^6^ The ability to anticipate which post–bariatric surgery patients are more likely to seek body contouring procedures can greatly benefit both patient and physician. Previous studies have investigated objective predictive factors by comparing post–bariatric surgery patients who underwent body contouring procedures and those who did not, but these reports are few and with inconsistent outcomes.^7^^-^9 The goal of our study was to prognosticate which post–bariatric surgery patients actually proceed to have body contouring procedures so that future patients meeting these traits can be targeted for education regarding plastic surgery.

## MATERIALS AND METHODS

This was a retrospective analysis undertaken with prior institutional review board approval. A total of 381 consecutive patients who underwent laparoscopic Roux-en-Y gastric bypass surgery at Yale New Haven Hospital between August 2002 and December 2005 were retrospectively reviewed. Selected patients were placed into 2 groups: group I consisted of all patients with subsequent body contouring procedures within the timeframe of the chart review; group II consisted of the remainder of the bariatric surgery patients without postoperative plastic surgery during this time period. Inclusion criteria included at least 1-year follow-up weight loss data. Noted body contouring procedures included panniculectomy, abdominoplasty, mastopexy, brachioplasty, and thigh lift. Objective variables studied were age, gender, preoperative excess body weight (EBW), percent excess weight loss (%EWL) at 6 and 12 months postoperatively, preoperative body mass index (BMI), and change in BMI from preoperative values at 6 and 12 months postoperatively. *Excess body weight* is defined as the difference between the patient's weight and the ideal weight taken from the 1983 Metropolitan Life Insurance Company data.^10^

Statistical analysis was performed using SPSS and Microsoft Excel. Significance of differences between groups was first assessed by univariate analysis of variance, followed by either the Student *t* test when assumptions for normality and equal variance were met, or Mann-Whitney *U* test where appropriate. Logistic regression was used to ascertain odds ratios to determine actual population probabilities for undergoing body contouring procedures. Unadjusted odds ratios were determined with univariate logistic regression. Adjusted odds for confounding, interactions, and best fit model were determined by multivariate logistic regression with manual stepwise reduction using the Hierarchy Principle and goodness-of-fit tests analysis.^11^ A result was considered statistically significant when *P* < .05.

## RESULTS

We identified 192 bariatric surgery patients who met inclusion criteria. Of this pool, a total of 24 patients underwent body contouring surgery with a mean follow-up of 749.2 ± 202.2 days (group I), whereas 168 did not with a mean follow-up of 850.8 ± 191.7 days (group II). Patients who declined body contouring surgery had a significantly longer length of follow-up with *P* value of .024. Mean age was a significant factor, group I mean age was 36 ± 9 years while group II was 41 ± 10 years (*P* = .023). Gender, preoperative BMI, and EBW distribution for each group were comparable and can be seen in Table 1. The mean time between bariatric surgery and the first body contouring procedure for group I patients was 538.5 ± 148 days.

At 6 months after bariatric surgery, there was only one variable that was statistically significant between the 2 groups—the change in BMI (*P* = .001). Patients who chose to undergo body contouring surgery decreased their initial BMI by 16.1 ± 4.0 kg/m^2^ and those who did not seek plastic surgery decreased their initial BMI by 13.8 ± 3.0 kg/m^2^. However, %EWL was not comparable between the 2 groups at the 6-month time point with group I %EWL of 54.4 ± 12.3 and group II %EWL of 50.31 ± 12.3 (*P* = .120). In addition, while the change in BMI from preoperative values was significantly different, comparison of the average 6-month BMI value itself was not. Group I had an average 6-month BMI of 36.7 ± 6.9 kg/m^2^ compared with 37.4 ± 7.6. kg/m^2^ for group II.

At 1 year postoperatively from bariatric surgery, both the change in BMI from preoperative levels and the %EWL were statistically significant (*P* < .001 and *P* = .024, respectively). Group I mean %EWL was 70.1 ± 13.3, whereas group II's mean %EWL was 62 ± 16.6. Group I mean change in BMI was 21.4 ± 6.6 kg/m^2^ compared with 17.4 ± 4.6 kg/m^2^ for group II. Again, while the change from preoperative BMI was significant, the average BMI for each group was not. Group I had an average BMI of 31.6 ± 4.9 kg/m^2^ compared with 34.1 ± 7.4 kg/m^2^ for group II.

Unadjusted odds ratios for undergoing plastic surgery for each independent variable of interest can be seen in Table 2. These values do not take into account confounding or variable interactions. Odds ratios for age, length of follow-up, change in BMI at 6 months, %EWL at 1 year, and change in BMI at 1 year were statistically significant. In the full model that accounts for interactions between all independent variables of interest, only age was statistically significant. During the process of elucidating the final model, %EWL at 1 year and change in BMI at 1 year were found to be confounding. The final model with the best goodness-of-fit determined age and the change in BMI at 1 year to be statistically significant predictors of undergoing body contouring procedures (Table 3). Patients 34 years of age or younger were 2.89 times more likely to undergo body contouring surgery, and patients were 1.124 times more likely to undergo body contouring surgery with each 1 kg/m^2^ increase in BMI difference at 12 months.

## DISCUSSION

Obesity is a multifaceted, complex disease that is managed by internists, endocrinologists, nutritionists, psychiatrists, bariatric surgeons, plastic surgeons, and other professionals. Given the multidisciplinary care necessary for optimum treatment of bariatric surgery patients, the American Society of Bariatric Surgeons advocates a center-of-excellence concept, which is currently being employed throughout the country. The center-of-excellence concept enables collaboration between the various clinicians at a single location.^1^ With close patient follow-up at these centers and a recommended 18-24 months' span between bariatric and plastic surgery, there is ample time with numerous opportunities for patient education on the risks and benefits of body contouring surgery.^12^^,^^13^ However, not every post–bariatric surgery patient will develop skin folds that warrant intervention. By targeting high-probability patients with additional body contouring–related information, surgeons can potentially prevent false expectations, improve patient satisfaction, improve consult efficiency, and increase the opportunity to finance the procedure; aspects previously shown to be obstacles for effective plastic surgery.^7^^,^^13^^-^17

Our results indicate predictive factors of age, %EWL, and postoperative change in BMI to assist in identification of suitable patients for post–bariatric surgery counseling about body contouring. We determined that patients were more likely to undergo plastic surgery if they were younger, had a shorter length of time since gastric bypass, a larger difference in BMI at 6 and 12 months, and a larger %EWL at 12 months. Pre–bariatric surgery weight conditions, such as initial BMI and EBW, were not correlated with having body contouring procedures. Weight gain functions through a similar mechanism on which long-term tissue expansion is based. Therefore in patients with a greater proportion of weight loss, the overlying skin will have lost more supporting elastic fibers and generated larger skin folds. As the size of these excessive skin folds increases, they are more likely to create functional impairment and compromise body image leading to the decision for surgery. Even though %EWL is the typical measure of success in evaluating bariatric surgery outcomes, the American Society for Bariatric Surgery also recommends using postoperative BMI change as a potential parameter.^10^ Thus, either measurement is readily available at 1 year and can serve as prognosticators for future body contouring surgery.

Our study also found that the mean time of follow-up for patients seeking body contouring surgery was shorter than follow-up for patients foregoing plastic surgery. Our finding is consistent with findings from previous studies that showed a shorter interval after bariatric surgery is associated with greater patient desire for plastic surgery, possibly because longer time elapsed leads to acceptance of excess skin or patients desiring plastic surgery would have had it earlier.^8^^,^^9^ This highlights the need to address patient education concerning body contouring surgery at an early stage.

Only one study in the plastic surgery literature to date has investigated predictive factors between patients who underwent plastic surgery and those who did not.^7^ Gusenoff et al analyzed 926 surveys from post–bariatric surgery patients and found that increased length of time since gastric bypass, a greater change in mean BMI, and incomes greater than $20,000 but not age to be significant objective prognosticators. In addition, they found that patients with greater changes in BMI are more likely to have body contouring procedures covered by insurance, suggesting the presence of medical necessity. This study was consistent with our finding that change in BMI is a predictable factor, though had contrary findings concerning patient age as a factor and length of follow-up since bariatric surgery. We believe these differences are attributable to differences in data collection methods, survey compared to retrospective review, and differences in the regional population of people visiting each institution.

Although our experience in this study provides several predictive factors for targeting patients to receive body contouring education, our report is subject to certain limitations. First, is the possibility that some patients may have been placed in the inappropriate group. The retrospective nature of this study does not account for patients in the non–body contouring surgery group who may have undergone plastic surgery at an outside facility, potentially skewing the data. We also acknowledge that our odds ratios of age and change in BMI at 12 months are not perfectly accurate in predicting the likelihood of patients undergoing body contouring procedures, and that patients outside of our follow-up period may still seek plastic surgery at a later time. Finally, we also recognize that the patient population who underwent bariatric surgery at this single institution may not be representative of the general post–bariatric surgery population. However, this study does provide predictive factors to more effectively target which patients should receive education on the benefits of body contouring surgery.

By improving patient education, plastic surgeons can diminish false expectations, improve patient satisfaction, improve consult efficiency, and increase affordability for the procedure. In this study, we demonstrated that the age of the patient, length of time since gastric bypass and the degree of changes in BMI at 6 and 12 months, and %EWL at 12 months can be used to help screen patients to determine who should be targeted more heavily with information regarding body contouring procedures. These measurements are convenient and available early in the post–bariatric surgery process.

## Figures and Tables

**Table 1:** Demographics for each group

	Mean ± SD		Analysis of Variance	***T* Test**
Variable	No Body Contouring	Body Contouring	*P*	*P*
Age (y)	41 ± 10	36 ± 9	.003	.003
Sex	82.8% female	83.3% female	.880	.880
EBW (lb)	176.20 ± 55.75	187.84 ± 61.2	.285	.285
Preoperative BMI (Kg/m^2^)	50.75 ± 8.68	52.42 ± 8.13	.318	.318
Follow-up (d)	850.8 ± 191.7	749.2 ± 202.2	.024	.024
Six-month BMI (Kg/m^2^)	37.36 ± 7.6	36.70 ± 6.9	.683	.683
Six-month change in BMI (Kg/m^2^)	13.82 ± 3.0	16.10 ± 4.0	.001	.001
Six-month %EWL (lb)	50.31 ± 12.3	54.40 ± 12.3	.120	.120
One-year BMI (Kg/m^2^)	34.10 ± 7.4	31.60 ± 4.9	.110	.110
One-year change in BMI (Kg/m^2^)	17.39 ± 4.6	21.40 ± 6.6	<.0001	<.0001
One-year %EWL (lb)	62.01 ± 16.6	70.11 ± 13.3	.024	.024

BMI indicates body mass index (kg/m^2^); EBW, excess body weight; %EWL, percent excess weight loss.

**Table 2:** Logistic regression univariate analysis for all variables of interest

Variable	Odds Ratio*	*P*
Age† (y)	0.955	.019
Age (<34 years)‡	3.113	.004
Preoperative BMI (kg/m^2^)	1.021	.319
EBW	1.003	.285
Follow up (d)	1.003	.027
Six month change in BMI (kg/m^2^)	1.221	.002
Six month %EWL (lb)	1.028	.118
One year BMI (kg/m^2^)	0.943	.110
One year change in BMI (kg/m^2^)	1.153	.001
One year %EWL (lb)	1.031	.026

BMI indicates body mass index (kg/m^2^); EBW, excess body weight; %EWL, percent excess weight loss.

*Odds ratio indicates odds per single unit increase.

†Continuous scale variable, in units of years.

‡Bivariate variable, with cutoff at 34 years of age.

**Table 3:** Logistic regression best-fit model

Variable	Odds Ratio*	*P*
Age (<34 years)†	2.891	.023
%EWL at 12 months (lb)	1.018	.241
One year change in BMI (kg/m^2^)	1.124	.009

BMI indicates body mass index; %EWL, percent excess weight loss.

*Odds ratio indicates odds per single unit increase.

†Age variable is bivariate, with cutoff at 34 years of age.
